# PRP and BMAC for Musculoskeletal Conditions via Biomaterial Carriers

**DOI:** 10.3390/ijms20215328

**Published:** 2019-10-25

**Authors:** Fabio S. M. Yamaguchi, Shahin Shams, Eduardo A. Silva, Roberta S. Stilhano

**Affiliations:** 1 Institute of Orthopedics and Traumatology, School of Medicine, University of São Paulo (USP), São Paulo 05403-010, Brazil; fsmy18@hotmail.com; 2 Department of Biomedical Engineering, University of California, Davis, CA 95616, USA; smshams@ucdavis.edu (S.S.); esilva@ucdavis.edu (E.A.S.); 3 Department of Physiological Sciences, Santa Casa de São Paulo School of Medical Sciences (FCMSCSP), São Paulo 01221-020, Brazil

**Keywords:** Platelet Rich Plasma (PRP), bone marrow aspirate concentrate (BMAC), biomaterials, muscle, bone, cartilage

## Abstract

Platelet-rich plasma (PRP) and bone marrow aspirate concentrate (BMAC) are orthobiologic therapies considered as an alternative to the current therapies for muscle, bone and cartilage. Different formulations of biomaterials have been used as carriers for PRP and BMAC in order to increase regenerative processes. The most common biomaterials utilized in conjunction with PRP and BMAC clinical trials are organic scaffolds and natural or synthetic polymers. This review will cover the combinatorial strategies of biomaterial carriers with PRP and BMAC for musculoskeletal conditions (MsCs) repair and regeneration in clinical trials. The main objective is to review the therapeutic use of PRP and BMAC as a treatment option for muscle, bone and cartilage injuries.

## 1. Introduction

Musculoskeletal conditions (MsCs) include several diseases that affect muscles, bones and cartilage [1]. MsCs are extremely prevalent and are responsible for more than USD 790 billion in health care costs per year in the United States alone [2]. Treatment of MsCs remains a challenging problem in traumatology, especially when there are volumetric losses and the damage exceeds the recovery capacity of the tissue.

Muscle injuries can occur through direct causes (e.g., lacerations, contusions, and strains) or indirect causes (e.g., ischemia, neurological dysfunction and hereditary myopathies) [3]. They are the most frequent cause of physical incapacity in sports practice [4]. Currently, the management of muscle injury is rest, ice, compression and elevation [4]. Anti-inflammatory medications [4,5], rehabilitation exercise programs [6,7,8,9], electrotherapeutic modalities, and hyperbaric oxygen therapy are used as well [10,11].

Bone injuries represent a challenging problem in traumatology. They entail a sustained increase in hospitalization, increased risk of complication, and associated increase in expense. The gold standard treatment for bone defect is the use of autologous bone graft [12]. However, it is associated with significant donor site morbidity and limited by the amount available for grafting, which has resulted in efforts to obtain biocompatible bone substitutes.

Osteochondral lesions represent an important type of bone injury as they result in significant health problems and are a leading cause of disability worldwide. Specifically, osteochondral lesions are defects on cartilage surfaces and are often related to traumatic origin (joint dislocation, ligament tear, meniscus tear, and fall/impact) [13,14,15]. The biomechanical properties of hyaline cartilage are easily compromised by traumatic injuries and cartilage has poor healing ability [16]. Thereby, the lesion may be irreparable and lead to chronic symptoms and early osteoarthritis (OA) [17]. Currently, treatments involve surgical procedures (chondroplasty, microfracture and spongialisation) or transplantation with an autograft or allograft. When the cartilage is severely damaged, a surgical procedure is necessary to replace the damaged tissue with a prosthetic device [14,15]. Despite all these advances in orthopedic field, the treatment for cartilage injuries remains challenging.

Musculoskeletal tissue engineering has been viewed as a viable alternative to the conventional therapies for muscle, bone and cartilage injuries. One of the goals of musculoskeletal tissue engineering is the delivery of cells or bioactive molecules to the tissue in order to increase tissue regeneration and repair [13]. Many types of biomaterials have been developed with this purpose and many of them have already been used in combination with platelet-rich plasma (PRP) and bone marrow aspirate concentrate (BMAC).

PRP and BMAC can be seen as orthobiologic therapies composed by cells, growth factors and bioactive molecules important to promote musculoskeletal tissue regeneration [18]. A growing number of pre-clinical and clinical trials have demonstrated the efficacy of PRP to treat muscle, bone and cartilage injuries. However, the use of PRP in orthopedics is still limited, especially due a lack of standardization of platelet-separation techniques [19]. The use of BMAC in clinical trials is recent when compared to PRP, however, good to excellent outcomes have been reported with BMAC, principally to treat cartilage injuries [18].

This review will cover the outcomes from clinical trials with PRP and BMAC and the combinatorial strategies of biomaterial carriers for MsCs treatment. The results from clinical trials will be summarized and discussed. Different preparations of PRP will be classified and explained. In a similar manner, the BMAC properties will be explained and the results from clinical trials will be discussed. Finally, the expansive literature on PRP, BMAC and biomaterials will be condensed to where these two fields meet for MsCs.

## 2. Platelet-Rich Plasma (PRP)

The first definition of PRP was a volume of autologous plasma with a primary target on platelet concentration above the baseline [20]; nonetheless, many other definitions have been proposed since then. PRP is a source of growth factors and has been used in hundreds of pre-clinical and clinical trials; however, the use of PRP is still controversial in orthopedics, especially due a lack of standardization of platelet-separation techniques [19]. Delong et al., proposed the PAW classification system: P—absolute number of platelets, A—activation methods and W—presence or absence of white cells [21]. PAW classification was very useful, however, it did not include the method used to prepare the PRP. In 2018, Harrison et al. proposed a new classification incorporating all previous classification systems, furthermore, including the preparation method. This new classification is based in the presence (L-PRP) or absence of leukocytes (PRP). The type of activation: I—without activation, II—activated, III—frozen-thawed preparations. The system used to prepare the PRP or PRF (platelet rich fibrin): 1-gravitational centrifugation, 2-Standard cell separators, 3-Platelet-pheresis. The concentration of platelets: A—platelet count of <900 × 10^3^/µL, B—platelet count of 900–1700 × 10^3^/µL, C—platelet count of >1700 × 10^3^/µL [21]. This classification system is important to more accurately compare protocols and results and we will use this system to classify all the PRPs used in this review.

PRP has been extensively tested in many pre-clinical and clinical trials for muscle, bone and cartilage, a compilation of the data obtained in said clinical trials is resumed in Table 1. According to the clinical trials website (www.clinicaltrials.gov) there exists a total of 48 trials for muscle, 40 for bone and 18 for cartilage. For muscle injuries, the efficacy of PRP remains unclear. Hamilton et al. performed a randomized, double-blind study with placebo group and evaluated the effect of PRP injections in athletics. The PRP was classified as Red-L-PRP-IIB2, according to Harrison et al. classification [22] (Table 1). However they did not observe effect in the rehabilitation of the athletes when compared with the controls group [23]. Similar results were also obtained by Reurink et al., they used a PRP classified as PRP-IA2 to treat hamstring injuries and they did not observe benefit of intramuscular PRP injections compared with the control group [24].

Martinez-Zapata et al. studied the benefit of autologous PRP, classified as PRP-IIB3 for the treatment of muscle rupture with haematoma and found that PRP did not improve the time to healing compared to that in the control group [47]. Hamid et al., combined PRP, classified as Red-L-PRP-IIB2, and rehabilitation program. The authors showed the injection of PRP, combined with rehabilitation after hamstring injury was more effective than a rehabilitation program alone [47]. Bubnov et al. evaluated the effect of PRP guided by ultrasound after muscle injuries in athletics and they found a reduction of pain, an improvement in the physical recovery, and faster regeneration when compared with conventional conservative treatment [46]. However, in this study, the authors did not detail the protocol to prepare the PRP and they did not show the concentration of cells after centrifugation, therefore we could not properly classify the PRP used. Rossi et al. showed a shortened time to return to sports in the group injected with PRP, when compared to the group that performed just the rehabilitation program [48]. In this publication, we did not find information about the PRP preparation and we could not classify the type of PRP. Thus, for muscle injuries the efficacy of PRP remains unclear. Nevertheless, there is a need for further studies that suggest therapeutic efficacy and report detailed quantification of the cells after PRP production.

There are several studies using PRP to treat a variety of bone injuries, for example in the treatment of extraction socket [39,49], humeral shaft fractures [44], mandibular fractures [38,43], non-union fractures [40,42] and others. The majority of these studies did not quantify the number of platelets and leukocytes and it was difficult to classify the type of PRP (Table 1). However, several studies showed positive effects of PRP for bone healing. Marx et al. prepared a PRP, classified as Red-L-PRP-IIB1, to treat mandibular fracture and the authors have found that PRP increased the graft maturity index and the trabecular bone area [36]. Daif et al. also used a PRP classified as Red-L-PRP-IIB1 and obtained a similar result [38]. The results for non-union fractures treatment showed a higher cure rate, fracture-healing acceleration and less pain in the group treated with PRP compared to the control group [40,42]. Both PRP in these papers were classified as B (Table 1).

For cartilage injuries, the majority of the studies were designed to treat OA [28,30,31,33,50] and the publications showed very promising outcomes. The majority of the clinical assays for cartilage treatment quantified the number of platelets and leukocytes, thus it was possible to classify the PRP. Kon et al. showed in two different publications the greater and longer efficacy of PRP compared to hyaluronic acid (HA) to treat OA [26,27]. The PRP produced was classified as IIB1. Two different publications, the first one using a PRP classified as PRP-IIA1 and the second, Red-L-PRP-IIB1, showed that PRP alleviated symptoms in early knee OA [29,33]. However, Filardo et al. showed that PRP, also classified as Red-L-PRP-IIB1, did not provide a superior clinical improvement, when compared to HA [31]. For meniscus repair, Kaminski et al. published the outcomes from two clinical trials, showing the use of PRP, classified as IIB1, improved the rate of chronic meniscal tear healing and increased the meniscus repair. [34,35]. Thus, the use of PRP for cartilage seems very promising.

## 3. Bone Marrow Aspirate Concentrate (BMAC)

In 1966, Friedenstein et al. reported the existence of bone marrow cells capable of generating hematopoietic cells, fibroblastic reticular cells, and bone in vivo [51]. These cells were designated as mesenchymal stem cells (MSC) and were simply defined by the capacity to adhere to plastic substrates and to produce colonies with self-renewal and multipotency [52,53]. MSCs are multipotent stem cells and have been used in the orthopedic field due to their strong self-renewal capacity, combined with the potential to differentiate in chondrocytes, adipocytes, and osteocytes [54]. In addition, MSCs secrete chemokines, cytokines, growth factors, and anti-inflammatory molecules that promote the recovery of the injured tissue [55].

Bone marrow aspirate concentrate (BMAC) is a method based in the autologous bone marrow aspiration, followed by centrifugation in order to concentrate MSCs, hematopoietic stem cells (HSCs), growth factors, white blood cells and platelets [56]. The percentage of MSCs in BMAC vary from 0.001% to 0.01% of mononuclear cells after centrifugation [57]. However, BMAC serves as a powerful source of growth factors, including transforming growth factor–beta (TGF-b), platelet-derived growth factor (PDGF), bone morphogenetic protein (BMP)–2 and BMP-7, which are important due to their anabolic and anti-inflammatory effects [57].

Recently, BMAC has emerged as a new option of treatment for MsCs, especially for cartilage and bone injuries. For cartilage, the majority of the studies were designed to treat OA of the knees, however the results remain unclear. A summary of the studies using BMAC, according to the tissue type is summarized in Table 2. Kim et al. injected 41 patients with BMAC prepared with the SmartPReP2 (Harvest) [58]. The results showed an improvement in both pain and functionality, however the best results were obtained in early to moderate stages [58]. Similar results were observed by Themistocleous et al. in which the authors concentrated the bone marrow manually by centrifugation [59]. The patients were injected with BMAC and the postoperative results showed a significant reduction in the numerical pain scale (NPS) and an increase in the Oxford knee score (OKS) [59]. Unfortunately, both publications did not quantify the number of progenitor cells, thus it is hard to compare the outcomes with other publications. Shapiro et al., treated 25 patients, using the contralateral knee as a control [60,61]. The authors used the Magellan system (Arteriocyte) to prepare the BMAC and injected 3.4 × 10^4^ MSCs and 4.62 × 10^6^ HSC in the knee. However, no differences were observed between placebo and treated knee 6 months after treatment [60] and 12 months after treatment [61]. Two studies were designed to treat osteochondral lesions of the talus, both used juvenile allogenic chondrocyte implantation (JACI) and BMAC [62,63]. The first study compared the pre- to postoperatively outcomes [62]. The authors treated 46 patients and observed an improvement in the mean of the scores obtained with the Short-form 12-item, version 2 (SF-12v2) and Foot and Ankle Outcome Score (FAOS) questionnaires. Of the 46 patients in the study, 22 had postoperative magnetic resonance imaging (MRI) scans that could be scored. Magnetic Resonance Observation of Cartilage Tissue (MOCART) score was 46.8 [62]. Unfortunately, this study did not quantify the cells and did not mention the system used to prepare the BMAC. In the second study, the authors used the Magellan system to produce the BMAC, however they did not quantify the number of progenitor cells [63]. The authors compared the microfracture (MF) with JACI–BMAC. Both treatments showed significant pre- to postoperative improvements in all FAOS subscale. However, MF showed a significant improvement in visual analog scale (VAS) and the average osteochondral lesion diameter was significantly larger in JACI-BMAC group compared to MF group. Thus, the authors concluded that BMAC did not result in significant gain as compared to MF [63].

For bone injuries, BMAC was used to treat nonunions and osteonecrosis of the femoral head (ONFH). The nonunion is defined as a nonprogressive healing proved by radiography during a period of 3 to 9 months since injury, without callus formation [69]. The non-union occurs in 5% to 10% of the injuries and the risk of nonunion is associated with necessitating surgical intervention [70]. ONFH is a prevalent disease originated by multiple causes and the main symptoms are severe pain, loss of movement and arthritis of the hip due to the femoral head collapse [67]. In 80% of cases a total hip replacement (THR) is necessary [68].

Herningou et al. evaluated 116 patients with ONFH that were treated with BMAC after core decompression with a small trocar [65]. The BMAC was prepared using the cell separator (Cobe 2991), 400× g/5 min. The mean of colony-forming units (CFU-F) obtained was 25 × 10^3^ cells in the BMAC after concentration [65]. The authors had to replace 34 hips (22 patients) among 189 hips treated. They observed better outcomes in patients with a higher number of progenitor cells transplanted [65]. Tabatabaee et al. randomized 28 hips in 2 groups: A- core decompression with BMAC, and B- core decompression without BMAC [67]. The bone marrow was filtered and washed. Then was centrifuged for 400× g/5–10 min. The mean of nucleated cells (NC) injected was 4.75 × 10^3^. The mean Western Ontario and McMaster Universities Osteoarthritis Index (WOMAC) and VAS scores in all patients improved significantly (*p* < 0.001). MRI showed a significant improvement in group treated with BMAC (*p* = 0.046) and significant worsening in the non-treated group (*p* < 0.001) [67]. Hauzeur et al. used the Spectra cell separator (777,006,300; Cobe) to produce the BMAC [68]. The authors performed a double-blind randomized control trial (RCT) to compare two groups: core decompression plus saline injection or core decompression plus BMAC implantation. Both groups included 19 patients (23 hips). The BMAC injected was composed by 3.46 × 10^9^ NC with CFU-F of 3.46 × 10^6^ NC. No differences were observed between groups for total hip replacement (THR) requirements, clinical evaluation, and radiological evolution. In both groups, 15/23 hips needed THR [68].

BMAC showed very promising results for cartilage and bone injuries, however more studies are necessary to better understand the real effect of BMAC. The number of progenitor cells is correlated with positive outcomes, however, obtaining a high number of cells depends on several factors like age of the patient, the method used to prepare the BMAC and the association with a biomaterial. The use of PRP and BMAC with biomaterials will be covered in the next topic.

## 4. Biomaterials

Broadly defined, a biomaterial is a synthetic or biologically derived material adapted for biomedical applications [71]. These materials are commonly used in many medical applications, including cardiovascular, orthopedic, and musculoskeletal tissue engineering [71]. Due to the innate differences between tissue types, a variety of materials have been utilized as biomaterials. The most common biomaterials utilized in conjunction with PRP and BMAC clinical trials are organic scaffolds and natural or synthetic polymers.

Organic scaffolds, such as bone chips, are used frequently in tissue-regeneration applications as these structures are thought to better recapitulate the local tissue environment in terms of structure and signaling capabilities, allowing for better integration of the implanted material into existing tissue architecture. These scaffolds can be cultured in or allowed to absorb bioactive molecules and be seeded with cells prior to application to the target site to expedite wound healing. For example, autologously isolated bone tissue can be directly implanted in the target region with little to no manipulation or can be decellularized and lyophilized to create an empty scaffold that, though devoid of cells, maintains the native bone extracellular matrix (ECM) structure.

Polymers account for more than half of the biomaterials currently on the market and can be synthetically generated or naturally sourced [71]. Due to the variety of sources and formulations polymer biomaterials have, they can take many forms for broad regenerative applications. Polymer materials have physical states ranging from soft hydrogels for applications in targeted controlled release of therapeutic molecules to rigid scaffolds for applications in tissue engineering [72,73,74,75]. Polymeric biomaterials vary widely in their physical properties, many of which can be tuned allowing them to be compatible with many tissue types including bone, cartilage, and muscle [73,76,77,78,79,80]. Scaffold like biomaterials are promising for applications in macro tissue regeneration, but lack of cell infiltration, proliferation, and differentiation inside these biomaterials have limited the benefits of their usage in regenerative medicine [77].

In order to address these problems, the addition of PRP or BMAC to biomaterial scaffolds and gels has been looked at as possible solution. Biomaterials are soaked with PRP with the goal to supply localized growth factors that will facilitate cell infiltration, proliferation, and differentiation [81]. Including BMAC to biomaterials is done in an effort to increase multipotent cell levels that can differentiate into local cell types and improve the health of the target region [82].

The most common biomaterials utilized with both PRP and BMAC in clinical applications are the natural biomaterials collagen and hyaluronic acid, and the synthetic biomaterial poly(lactic-co-glycolic) acid.

### 4.1. Collagen

Collagen is the primary structural component of the ECM, and is therefore also one of the most largely studied polymer structures for tissue regeneration due to its biocompatibility, biodegradability, and cell interactivity [83]. Collagen molecules are made of three polypeptide chains aligned in a parallel manner, coiled, and stabilized by h-bonds between strands which are capable of forming macroscopic fibrous networks [83]. Collagen networks are highly supportive of cell infiltration and can interact with cells via receptors, or through secondary interactions with collagen associated proteins such as decorin, laminin, and other RGD(Arg-Gly-Asp) -containing proteins [83]. A third of all protein in humans is collagen based, and over thirteen types of collagen have been identified, though the most used in biomaterial applications is collagen type 1 which is found in skin, tendon, and bone [84]. Due to the variety of tissues collagen is found in, it has been used in tissue regeneration efforts in many tissue types, including bone, muscle, and cartilage. Collagen scaffolds have also been shown to guide differentiation of tissue specific cell types with the addition of PRP, and have promoted tissue regeneration and remodeling by bone marrow associated cells, lending credence to its use in clinical PRP and BMAC applications [85,86].

### 4.2. Hyaluronic Acid (HA)

HA is a naturally occurring glycosaminoglycan found in the extracellular matrix of animal tissues, and has been used in a variety of tissue engineering applications as a bare scaffold material, a drug delivery device, and a cell seeded construct [87]. Predominantly found in the eye and in cartilage tissues, HA is biocompatible and cell adhesive and, therefore, it is able to interact with cellular proteins to allow for cell infiltration. In addition, HA is tunable to support a variety of structural designs ranging from single layered highly organized bioprinted layouts, to micropatterning techniques, to macroscale ECM-simulating scaffolds [87,88,89]. HA scaffolds can be engineered to contain specific biodegradation kinetics that allow for enhanced control over bioactive molecule delivery, and have been shown to enhance proliferation and protein secretion of specific cell types [90]. The addition of PRP to scaffolds that contain HA has shown that induction of differentiation of chondrocytes is possible, and thus is very promising in fields focusing on cartilage tissue regeneration [91]. Due to the large amounts of HA in native cartilage tissue and its ability to maintain if not promote chondrocyte activity, many BMAC clinical applications have utilized HA materials, and have shown strong applicability in macroscale defect cartilage tissue engineering. In addition, HA and other saccharide-containing material platforms are continuing to show promise in expanding the applicability of PRP and BMAC therapies for tissue regeneration [92,93].

### 4.3. Poly (Lactic-Co-Glycolic) Acid (PLGA)

Poly(lactic-co-glycolic) acid (PLGA) is a synthetic copolymer substance frequently used in tissue engineering and drug delivery applications [94]. PLGA is biocompatible, biodegradable, and has tunable physical and mechanical properties for applications in a variety of tissue types. This tunability is influenced mainly by altering the ratio of the two monomers polylactic acid (PLA) and polyglycolic acid (PLG), which varies the amount of ester linkages and thus the points subjected to degradation by hydrolysis [94,95,96]. PLGA has been approved for a variety of drug delivery applications, and is able to be chemically modified to improve cell adhesive qualities for applications in tissue regeneration and cell therapies [94,97,98]. PLGA can also be used in blended polymer formulations with either natural or synthetic materials to increase cell interactive properties. PRP and BMAC loaded PLGA scaffolds have shown promise in multiple tissue type regeneration studies, including models focusing on cartilage and bone [99,100,101,102]. In addition, clinical applications of PRP and BMAC loaded into PLGA scaffolds have demonstrated significantly increased quality of repair by both quantitative and clinical evaluations in cartilage regeneration efforts [103,104,105].

Both PRP and BMAC used in conjunction with biomaterials have shown promise in clinical trials to regenerate both bone and cartilage, as summarized in Table 3 and detailed below.

Bone regeneration clinical trials utilizing biomaterials in conjunction with PRP and BMAC range from naturally sourced scaffolds to inorganic slurries and have shown promising, although mixed, results. Regenerative therapy for osseous tissue often relies on bone grafting, the most common regenerative therapy for bone [108]. Grafts are a wide range of materials and include autologously isolated tissues from patients and alloplastic materials. Dallari et al. utilized lyophilized bone chips supplemented with PRP or PRP/BMAC to treat 33 patients undergoing high tibial osteotomy for genu varum [103]. Patients were split into three groups–A, B, and C—that received bone chips with PRP, bone chips with PRP and BMAC, or empty bone chips, respectively [103]. Clinical evaluation utilizing the Knee Society scoring system and radiography were used to define outcome at six weeks, 12 weeks, six months, and one year. As early as six weeks following the procedure, histomorphometry showed significantly higher numbers of bone forming cells in groups A and B compared to the control group C [103]. Radiographically, groups A and B showed significantly higher osteointegration at all time points when compared to controls, and group B showed significantly higher osteointegration that group A until the six-month time point [103]. At a full year, all groups had relief of knee pain and reported improvements in walking ability; in addition, all patients showed complete healing at a clinical and functional level regardless of group [103].

Biomaterials isolated from bone, such as hydroxyapatite (HAp) have also been used with PRP and BMAC. HAp is the main mineral component of bone tissue, and it has been largely applied to clinical bone tissue engineering efforts with promising results [129]. One clinical study on endodontically induced periapical lesion repair using HAp scaffolds utilized HAp/PRP, HAp alone, PRP alone, and no intervention as the test group—the test group, two positive controls and a negative control, respectively [112]. This 20 patient study returned positive results as seen in complete bone regeneration in all treated groups with most rapid regeneration seen in the HAp/PRP group at 6 months, while PRP and HAp groups alone had complete regeneration at 9 months and 1 year, respectively [112]. Thus, though HAp scaffolds and PRP alone returned positive and complete regeneration, utilizing a combinatorial approach resulted in expediated healing [112]. This trend is supported by other studies, such as Okuda et al., which utilized HAp loaded with PRP to treat osseous defects in 70 patients with chronic periodontitis. Thirty-five subjects were treated with HAp/PRP, and 35 were treated with HAp/saline as a control [111]. Clinical outcomes at one year post-treatment were evaluated by probing depth (PD), clinical attachment level (CAL), gival recession (GR), while quality of repair of the defect fill was determined by radiography [111]. Clinical evaluation for the HAp/PRP group after a year showed significant improvement compared to the control in all parameters [111]. Although clinical evaluations were promising, radiographic defect fill was not significantly different between groups [111]. In contrast, Menezes et al. demonstrated that after 4 years, that Hap/PRP had significantly better clinical outcomes (probing depth reduction and clinical attachment gain) and quality of repair as determined by radiographic determination of defect fill in periodontal intraosseous defect model compared to HAp/saline controls [113]. BMAC alone has also been utilized with HAp scaffolds. Jäger et al. investigated BMAC application in HAp or collagen scaffolds in a human bone defect model of 39 patients [104]. Clinical evaluation was determined by mobility and activity pre and post intervention, and quality of repair was determined radiographically [104]. Although both groups showed complete bone healing, the HAp treated group of 27 patients showed significantly earlier and complete bone formation compared to the collagen sponge group—suggesting that scaffold choice can impact the rate of therapeutic benefit BMAC can provide [104].

Synthetic materials have also been studied as scaffolds for bone regeneration. Calcium sulfate products, such as medical grade calcium sulfate hemihydrate (MGCSH), have a long history of use in regenerative efforts. Calcium sulphate was one of the first bone substitutes applied in orthopedics and dentistry because it was easily sterilized, inexpensive, and biocompatible [130]. When mixed with PRP, MGCSH scaffold group showed significantly higher vital bone after 3 months when compared to a collagen plug control in a tooth extraction socket model [114]. β-Tricalcium phosphate (β-TCP) is an osteoconductive matrix that promotes bone remodeling, therefore, because of these features, β-TCP is a promising bone grafting material [131,132]. β-TCP has been used extensively in conjunction with PRP in clinical bone regeneration efforts, with mixed results. Intrabony defects were treated with β-TCP or β-TCP/PRP in a clinical study of 14 chronic periodontitis patients and returned no significant difference between groups both clinically and radiographically after 6 months [110]. Similarly, no significant differences were seen between groups (β-TCP, β-TCP + PRP, β-TCP + PRP + membrane) clinically or radiographically in a 25-patient, interproximal intrabony defects clinical study after a year post intervention [107]. In contrast, Saini et al. showed significant differences in clinical evaluations (probing pocket depth and clinical attachment level) and linear bone fill between β-TCP and β-TCP + PRP groups after 9 months in a 20-patient bilateral infrabony defect model [109]. Attia et al. also returned significantly better improvements both clinically and radiographically in a PRP/β-TCP group compared to β-TCP control in an 18-patient 1-year postoperative follow-up intrabony defect clinical study [108].

### 4.4. Cartilage

Osteochondral (cartilage) tissue engineering advances rely on the fabrication of biomaterial scaffolds due to the limited ability of cartilage to regenerate and self-repair [133]. Clinical trials for cartilage healing and regeneration using biomaterials with PRP or BMAC utilize a different variety of materials than bone. While bone is rigid and motivates the use of stiffer and mineralized scaffolds, cartilage is composed of ECM rich in collagen and is up to 80% tissue fluid by weight [134]. Therefore, materials commonly used in cartilage tissue engineering are polymers, since polymers can be engineered to have similar physical properties of native cartilage tissue. Due to cartilage consisting largely of collagen, collagen has been widely studied in clinical trials with PRP and BMAC therapies in efforts to regenerate cartilage. Dhollander et al. utilized a collagen I/III scaffold with platelet-rich plasma to treatment of osteo chondral patellar legions in the knee in 5 five patient pilot clinical trial [123]. Clinical evaluations on VAS, the Knee injury Osteoarthritis Outcome Score (KOOS), Tegner activity scale, and Kujala patellofemoral score were taken preoperatively, and at 1 and 2 year follow-ups postoperatively, which showed improvements [123]. MOCART scoring taken MRI data to confirm quality of repair showed complete integration with patient tissue, but irregularities in surface features and incomplete filling two years after intervention [123]. Therefore, though showing some improvement, no significant benefits can be stated. BMAC used with collagen returned more promising therapeutic results. In a 9-patient pilot study, focal lesions of condylar articular cartilage were treated with arthroscopic microfractures that were then covered in a collagen membrane immersed in BMAC showed significant clinical and repair benefit [124]. Clinical evaluations by the International Knee Documentation Committee (IKDC) score, Lysholm score, VAS, and activity levels differed significantly 1 year postoperatively compared to preoperative scores for all but one patient [124]. MRI scans taken 6–9 months postoperatively demonstrated original cartilage levels were reached despite irregularities and edema being present in all patients. However, despite these promising figures, when samples were taken and histologically analyzed from 4 of 9 patients, only a single patient had hyaline like cartilage formation while the remaining had some level of fibrocartilage present [124]. In addition, while all cartilage implants were determined to be reabsorbed in these 4 cases, cell arrangements in the bioptic samples were unlike that of native cartilage tissue [124]. Another microfracture, BMAC, and collagen membrane case study, in this case to treat cartilage trauma, showed beneficial clinical improvements in patient activity and mobility as early as 6 weeks postoperatively, and MRI scans taken a year following showed defect filling with no signs of bone marrow edema [82]. Collagen scaffolds have also showed positive trends, as demonstrated in a pilot study of 5 patients that received BMAC seeded into collagen scaffolds for chondral articular defects [125]. Clinical evaluations relied on patient reports, which were asymptomatic at the 1-year postoperative level [125]. Quality of repair was determined by both visual examination and histologically. At the time of biopsy, the surgeon performing the procedure performed the standard ICRS (International Cartilage Repair Society) Cartilage Repair Assessment (CRA) and all 5 patient biopsies were categorized as nearly normal [125]. Upon histological examination, 1 patient had hyaline-like cartilage, 1 had fibrocartilage, and the remaining 3 had a mixture of hyaline/fibrocartilage, and all scaffolds had been reabsorbed [125].

Forty eight patients suffering from osteochondral lesions were treated with BMAC loaded in either collagen or hyaluronic acid scaffolds in a prospective clinical study in Giannini et al. (2009) [119]. Clinical outcome determined by American Orthopaedic Foot and Ankle Society (AOFAS) scores looked at the influence of scaffold type, lesion area, lesion depth, and previous surgical intervention—which showed improvement after 2 years, though not significantly [119]. MRI showed tissue regeneration, and histologically, biopsies also showed regeneration of cartilage tissue, though not entirely hyaline [119]. No significant differences were seen between collagen and hyaluronic acid scaffolds [119]. A midterm study by members of the same group looked exclusively at clinical outcomes of 64 patients suffering from osteochondral lesions via AOFAS scores, which demonstrated significant clinical improvement when treated with BMAC and either collagen or hyaluronic scaffolds [135]. Continuing this positive trend in this same model and scaffolds, Giannini et al. (2013) demonstrated significant clinical improvement by AOFAS score at 2, 3, and 4 years postoperatively [126]. MRI T2 mapping on 20 of the 49 participants in this clinical study showed statistically significant improvement and hyaline like cartilage formation, and MOCART scores illustrated 9 of the 20 patients had complete defect fillings and 13 of the 20 had integration at the border zone [126]. MOCART scores also demonstrated complications were also present in a minimum of 12 of 20 patients [126]. Together these three studies illustrate two different natural materials can result in clinically significant improvements. HA scaffolds delivering BMAC in osteochondral lesions of the knee have also been shown to be significantly beneficial clinically according to IKDC and KOOS in a 20 patient clinical study [120]. In addition, MRIs taken at 1 and 2 years postoperatively showed cartilage regeneration, which was supported by histological analysis that determined high levels of collagen II in a proteoglycan rich matrix [120].

Poly (glycolic acid)-hyaluronic acid (PGA-HA) composite material has also been extensively studied in clinical settings in conjunction with PRP and BMAC. In a 52 patient clinical study, PRP soaked PGA-HA implants showed clinically significant improvement at 1, 2 and 5 years postoperatively compared to baseline [81,115,116]. Histological analysis of biopsies taken at 2 years showed that tissues were rich in chondrocyte like cells, proteoglycans, and collagen II [82]. MOCART scoring at a 4 year follow-up showed complete cartilage repair in 20 of 21 patients—demonstrating lasting improvement in PRP therapies applied by PGA-HA implants. PRP-soaked PGA-HA applied to 45 patients with hallus rigidus after 3 stage resection arthroplasty showed clinically significant AOFAS and ROM and radiographical findings showed no anomalies 2 years postoperatively [117]. BMAC delivered by PGA-HA showed clinically significant IKDC score improvement [118]. In addition, MRI data showed complete defects filling was observed and surface appearance returned to previous cartilage level despite several defects [118].

## 5. Conclusions

PRP and BMAC are promising therapeutics for MsCs repair and regeneration. Clinical data have shown that PRP and BMAC are safe and showed positive results for both cartilage and bone injuries. There is a lack of studies comparing PRP and BMAC outcomes in bone and cartilage and there are no clinical trials using BMAC to treat muscle injuries. PRP has many advantages: (1) the method is economical, (2) the production does not require complex equipment, (3) the technique to collect is not invasive and (4) PRP has a low risk of immune response [136]. On the other hand, there is still no consensus regarding the techniques to prepare the PRP and the majority of clinical trials are not double-blind controlled with a large number of patients.

The use of BMAC in the orthopaedic field is still recent, compared to PRP, but the results are very promising. Although progenitor cells have been used for a long time in clinics, BMAC is not only composed by progenitor cells, but also has a large quantity of growth factors. This combination makes BMAC a powerful therapy. However, obtaining BMAC is invasive, requires closed systems during the preparation, and the positive results are strongly correlated with the number of stem cells.

Biomaterials have been combined with PRP and BMAC in order to localize and extend bioactive molecule effects and increase cell interactive properties. When designing a macroscale tissue-engineering solution to treat MsCs, it is essential to not only select the correct therapeutic, but also the most suitable biomaterial system for employing the therapeutic in vivo. Local tissue architecture varies vastly between musculoskeletal tissues, can vary broadly from person to person, and functions differently in pathological conditions—therefore, materials must be chosen to address all of these needs to maximize therapeutic outcomes of the deliverable. As PRP and BMAC come to the clinic, their physical properties must be taken into account in order to ensure their therapeutic potential is maintained. Cells are sensitive to physical and chemical properties of their local environment; therefore, the biochemical properties of the biomaterial carrier must be tuned to ensure that mechanical forces and signaling potential both within and outside the material carrier are within ranges that are sustainable by the local tissue and the delivered therapeutic [75]. If these conditions are not met, not only will therapeutic benefits be limited due to biochemical diffusivity limitations and delivered cell morbidity, but the injury itself may be exacerbated due to negative host–biomaterial interactions. Biomaterials that are engineered to mimic local, healthy signaling pathways and native mechanical properties are able to be incorporated within local tissue architecture and minimize abrasive host–biomaterial interactions [75,137]. Therefore, the application of a biomaterial is not only that of a therapeutic carrier of PRP and BMAC, but also as a functional regenerative scaffold for cell integration, proliferation, and differentiation that can expedite macroscale musculoskeletal tissue healing.

Future directions should address biomaterials that have been optimized to mimic local tissue properties and seek to incorporate PRP/BMAC with this level of engineering design to further improve tissue-regeneration effects.

## Figures and Tables

**Table 1:** Main findings of the use of platelet-rich plasma (PRP) in clinical trials according to the tissue type.

Tissue	Study	System	Anticoagulant	Classification *	Findings
**Cartilage**	Sanchez et al., 2008 [25]	Manual 640× g/8 min	Sodium citrate	II1	(+) significant improvement of pain
	Kon et al., 2010 [26]	Manual 1800 rpm/15 min 3500 rpm/10 min	Sodium citrate	IIB1	(+) reduction of pain and improvement of knee function in younger patients with low degree of articular degeneration
	Kon et al., 2011 [27]	Manual 1480 rpm/6 min 3400 rpm/15 min	DNS	IIB1	(+) PRP greater and longer efficacy than HA injection
	Lee et al., 2013 [28]	Magellan Autologous Platelet Separator (Medtronic Biologic Therapeutics and Diagnostics)	Sodium citrate	IB2	(+) significant improvement in clinical results in early OA.
	Patel et al., 2013 [29]	Manual 1500 rpm/15 min Leukocyte filtered	Citrate phosphate dextrose	PRP-IIA1	(+) effective to alleviate symptoms in early knee OA
	Duif et al., 2015 [30]	ACP-system (Arthrex) 1500 rpm/5 min	DNS	2	(+) pain reduction, gain knee function
	Filardo et al., 2015 [31]	Manual 1480 rpm/6 min 3400 rpm/15 min	DNS	Red-L-PRP-IIB1	(=) PRP do not provide a superior clinical improvement when compared to HA
	Sanchez al, 2016 [32]	Manual 580× g/8 min	Sodium citrate	PRP-IIA1	(+) multiple injections of PRP are useful in achieving better clinical results in early OA
	Gormeli et al., 2017 [33]	Manual 1500 rpm/6 min 3500 rpm/12 min	DNS	Red-L-PRP-IIB1	(+) multiple injections of PRP are useful in achieving better clinical results in early OA
	Kaminski et al., 2018 [34]	DNS	DNS	II	(+) increased meniscus repair
	Kaminski et al., 2019 [35]	Manual 900 rpm/9 min 3200 rpm/15 min	DNS	IIB1	(+) significant improvement in the rate of chronic meniscal tear healing
**Bone**	Marx et al., 1998 [36]	Manual 5600 rpm and 2400 rpm	Citrate Dextrose	Red-L-PRP-IIB1	(+) enhanced bone graft in mandibular fracture
	Rodriguez et al., 2003 [37]	Smart Prep (Harvest Technologies)	DNS	II2	(+) effective in maxillary sinus augmentation
	Daif et al., 2012 [38]	Manual 1200 rpm/20 min 2000 rpm/15 min	Sodium citrate	Red-L-PRP-IIB1	(+) enhanced bone regeneration in mandibular fracture
	Anitua et al., 2015 [39]	Manual 580× g/8 min	Sodium citrate	I1	(+) enhanced healing of extraction socket
	Malhotra et al., 2015 [40]	DNS	DNS	PRPIB	(+) fracture healing acceleration in nonunion fractures
	Tabrizi et al., 2015 [41]	Manual 800× g/5 min 1500× g/5 min	Citrate phosphate dextrose	II1	(+) enhanced bone formation in bone cavity
	Ghaffarpasand et al., 2016 [42]	Gravitational Platelet Separation System (GPS III; BIOMET) 3200 rpm/15 min	Acid-citrate dextrose	B2	(+) higher cure rate and less pain in non-union fractures
	Castillo-Cardiel et al., 2017 [43]	PRGF System III (BTI) 450× g/8 min	Sodium citrate	II2	(+) increase of bone intensity and density in mandibular fractures
	Acosta-Olívio et al., 2017 [44]	Manual 1800 rpm/5 min 3200 rpm/3 min	Sodium citrate	II1	(+) earlier bone consolidation in shaft fractures
**Muscle**	Hamid et al., 2014 [45]	GPS III	DNS	Red-L-PRP-IIB2	(+) PRP combined with rehabilitation program was more effective in treating hamstring injuries than rehabilitation program alone
	Hamilton et al., 2015 [23]	GPS III 3200 rpm/15 min	Citrate dextrose	Red-L-PRPIB2	(−) no benefit of PRP injection compared to rehabilitation in athletes
	Reurink et al., 2015 [24]	ACP-system	EDTA	PRP-IA2	(−) no benefit of PRP injections compared with placebo in patients with acute hamstring
	Bubnov et al., 2016 [46]	Manual DNS	DNS	1	(+) injections of PRP under ultrasound guidance had higher level of pain relief, physical recovery, and faster regeneration compared with conventional conservative treatment in acute muscle trauma in professional athletes
	Martinez-zapata et al., 2016 [47]	Platelet apheresis	DNS	PRP-IIB3	(−) PRP did not improve the time to healing compared to that in the control group
	Rossi et al., 2017 [48]	Manual 1400 rpm/3 min 3000 rpm/4 min	EDTA	I1	(+) PRP injection combined with a rehabilitation program me shortened time to return to sports compared to a rehabilitation programme only

(+) superior results, (−) inferior results, DNS = data not shown, HA = hyaluronic acid, OA = osteoarthritis. * PRP was classified according to DeLong et al. [21].

**Table 2:** Main findings of the use of bone marrow aspirate concentrate (BMAC) in clinical trials according to the tissue type.

Tissue	Study	BMAC preparation	# Progenitor Cells Injected	Follow-up	Findings
**Cartilage**	Kim et al., 2014 [58]	SmartPReP2 Bone Marrow Procedure Pack BMAC2 kits (Harvest Technology)	DNS	12 months	(+) 41 patients injected with BMAC. VAS showed significant pre- to postoperative improvement. All functional scores were increased after the procedure. Better outcomes were obtained in early to moderate stages of OA than more advanced stages. (OA of the knees)
	Shapiro et al., 2016 [60]	Filtered 170 µm Magellan Autologous Platelet Separator System (Arteriocyte)	MSC = 3.44 × 10^4^ HSC = 4.62 × 10^6^	6 months	(=) 25 patients, 13 were injected with BMAC in their right knee and placebo in the left knee and 12 received the opposite. No differences were observed between placebo and treated knee. (OA of the knees)
	Desandis et al., 2017 [64]	DNS	DNS	16.7 months	(+) 46 patients treated with JACI–BMAC were retrospectively evaluated. The mean questionnaire SF-12v2 and FAOS improved significantly from pre- to postoperatively. Of the 46 patients in the study, 22 had postoperative MRI scans that could be scored. MOCART score was 46.8. (Osteochondral Lesions of the Talus)
	Shapiro et al., 2018 [61]	Filtered 170 µm Magellan Autologous Platelet Separator System (Arteriocyte)	MSC = 3.44 × 10^4^ HSC = 4.62 × 10^6^	12 months	(=) 25 patients, 13 were injected with BMAC in their right knee and placebo in the left knee and 12 received the opposite. BMAC did not show superior results compared to saline group. (OA of the Knees)
	Themistocleous et al., 2018 [59]	2800 rpm/15 min	DNS	11 months	(+) 121 patients treated with BMAC were retrospectively evaluated. NPS decreased 8.33 preoperatively to 4.49 postoperatively (*p* < 0.001). The mean Oxford knee score (OKS) increased from 20.20 pre-operatively to 32.92 postoperatively (*p* < 0.001). (OA of the Knees)
	Karnovsky et al., 2018 [63]	Magellan Autologous Platelet Separator (Anteriocyte Medical Systems)	DNS	28.1 months	(−) 30 patients treated MF and 20 who received JACI-BMAC were retrospectively evaluated. Both treatments showed significant pre- to postoperative improvements in all FAOS subscale. MF showed a significant improvement in VAS. Average osteochondral lesion diameter was significantly larger in JACI-BMAC group compared to MF group. (Osteochondral Lesions of the Talus)
**Bone**	Hernigou et al., 2002 [65]	Cell separator (Cobe 2991) 400× g/5 min	CFU-F = 25 × 10^3^ cells	7 years	(+) 116 patients (189 hips) injected with BMAC after core decompression with a small trocar. Total hip replacement was needed in 34 hips (22 patients) among 189 hips treated. Patients with a higher number of progenitor cells transplanted had better outcomes (ONFH)
	Hernigou et al., 2005 [66]	Cell separator (Cobe 2991) 1200× g/5 min	CFU-F = 5.1 × 10^4^ cells Progenitors = 5.49 × 10^4^ (53 patients) 1.93 × 10^4^ (7 patients)	4 months	(+) Bone union was obtained in 53 of the 60 patients that received the higher number of progenitor cells. The BMAC efficacy is related to the number of progenitors in the graft. (Nonunions)
	Tabatabaee et al., 2015 [67]	Bone marrow was filtered and washed. Then was centrifuged for 400g/5–10 min	NC = 4.76 × 10^3^ cells	24 months	(+) 28 hips were randomized in 2 groups of core decompression with and without BMAC. The mean WOMAC and VAS scores in all patients improved significantly (*p* < 0.001). MRI showed a significant improvement in group treated with BMAC (*p* = 0.046) and significant worsening in the non-treated group (*p* < 0.001). (ONFH)
	Hauzeur et al., 2018 [68]	Spectra cell separator (777,006,300; Cobe)	NC = 3.46 × 10^9^ cells CFU-F = 3.46 × 10^6^ NC	24 months	(=) Double blind RCT study comparing two groups: core decompression plus saline injection or core decompression plus BMAC implantation. Both groups included 19 patients (23 hips). No differences were observed between groups for THR requirements, clinical evaluation and radiological evolution. In both groups, 15/23 hips needed THR. (ONFH)

(+) superior results, (−) inferior results, (=) similar results, CFU-F: colony-forming units, DNS = data note shown, HSC = hematopoietic stem cells, FAOS = Foot and Ankle Outcome Score, JACI = juvenile allogenic chondrocyte implantation, MF = microfracture, MOCART = Magnetic Resonance Observation of Cartilage Tissue, MSC = mesenchymal stem cells, NC = nucleated cells, NPS = numerical pain scale, OA = osteoarthritis, OKS = Oxford knee score, ONFH = osteonecrosis of the femoral head, RCT = randomized controlled trial, SF-12v2 = Short-form 12-item, version 2, THR = total hip replacement, VAS = visual analog scale, WOMAC = Western Ontario and McMastern Universities Osteoarthritis Index.

**Table 3:** Main biomaterials used with PRP and/or BMAC to treat cartilage and bone injuries.

Tissue	Study	Biomaterial	Formulation	Preparation (PRP/BMAC)	Follow-up	Findings
**Bone**	Dallari et al., 2007 [103]	Bone Chip	Scaffold	PRP/BMAC	1 year	(=) No significant difference between PRP/BMAC groups and empty lyophilized bone controls as all patients reported relieved knee pain and full range of motion.
	Sauerbier et al., 2010 [106]	Bovine Bone Mineral	Particles	BMAC	4 months	(=) New bone formation was 19.9% but not significantly different from the synthetic polysaccharide isolation method control.
	Jager et al., 2011 [104]	Collagen	Sponge	BMAC	1 year	(+) Radiography showed significant bone remodeling in all groups, but healing was longer when compared to BMAC/hydroxyapatite controls.
	Yassibag-Berkman et al., 2007 [107]	β-TCP	Slurry	PRP	1 year	(=) No significant difference between clinical parameters in PRP and control groups
	Attia et al., 2010 [108]	β-TCP	Slurry	PRP	1 year	(+) Significant reduction of probing depth and increase in clinical attachment gain (*p* < 0.01)
	Saini et al., 2011 [109]	β-TCP	Slurry	PRP	9 months	(+) Significant decrease in pocket depth and increase in clinical attachment (*p* < 0.05) in β TCP/PRP compared to β TCP alone
	Özdemir et al., 2012 [110]	β-TCP	Slurry	PRP	6 months	(=) All 6 parameters evaluating clinical outcome were not significant between PRP and control groups (*p* < 0.05)
	Okuda et al., 2005 [111]	HAp	Scaffold	PRP	1 year	(+/−) There were significant differences in gingival index, bleeding on probing, probing depth and clinical attachment level, in PRP groups compared baseline (*p* < 0.001), and significant differences in PRP versus control groups in probing depth, clinical attachment gain, and vertical attachment (*p* < 0.05) No significant difference in defect change between PRP scaffolds versus saline scaffolds, however, positive significant gain was seen compared to baseline measurements (*p* < 0.01)
	Vaishnavi et al., 2011 [112]	HAp	Scaffold	PRP	1 year	(=) Radiographic evaluation showed bone regeneration in all groups (scaffold, PRP, scaffold with PRP) except the negative control
	Menezes et al., 2012 [113]	HAp	Scaffold	PRP	4 years	(+) Significant differences in defect fill were seen in PRP/hydroxyapatite treated group compared to hydroxyapatite/saline control (*p* < 0.001)
	Kutkut et al., 2012 [114]	MGCSH	Scaffold	PRP	3 months	(+) Radiographic evaluation confirmed more dense bone in MGCSH and PRP groups compared to empty MGCSH, and histomorphic analysis showed a statistically significant difference between these groups (*p* < 0.05)
**Cartilage**	Siclari et al., 2012, 2014 [81,115,116]	PLGA-HA	Matrix	PRP	1–5 years	(+) Histology showed homogenous repair tissues and good integration of repair tissues to the subchondral bone and adjacent cartilage and immunohistochemistry showed signs of hyaline like cartilage formation. At 2 years, hyaline-to hyaline cartilage repair tissue that was rich with a chondrocyte morphology, proteoglycans, and type-II collagen. At 4 years, MRI confirmed good defect and volume filling in 20 of 21 patients and received high MOCART scores.
	Siclari et al., 2018 [117]	PLGA-HA	Matrix	PRP	2 years	(+) AOFAS rating increased significantly (*p* < 0.01). ROM increased significantly (*p* < 0.01).
	Enea et al., 2013 [118]	PLGA-HA	Matrix	BMAC	2 years	(+) MRIs showed all patients had complete defect and volume filling and resurfacing of articular cartilage to previous cartilage level. Some bone marrow edema and subchondral irregularities were observed—as well subchondral irregularities.
	Giannini et al., 2009 [119]	HA	Membrane	BMAC	2 years	(+) MRI done 12 months postoperatively showed tissue regeneration in all 48 patients. Integration with the healthy cartilage was complete, and transition zones were smooth in all patients. Immunohistologic results confirmed new cartilaginous tissues with varied hyaline cartilage tissue remodeling.
	Buda et al., 2010 [120]	HA	Scaffold	BMAC	2 years	(+) Post-treatment IKDC and KOOS scores were significantly higher than pre-treatment (*p* < 0.0005). MRIs taken at 1 and 2 years after treatment shown subchondral bone and cartilage regeneration. Histological analysis showed a proteoglycan rich matrix and collagen II throughout the regenerated tissue.
	Gobbi et al., 2016 [121]	HA	Scaffold	BMAC	5 years	(+) All HA-BMAC scaffold treated patients maintained classification as normal or nearly normal by IKDC, KOOS, Tegner, and Lysholm. No quality of repair studies were done in this study.
	Gobbi et al., 2017 [122]	HA	Scaffold	BMAC	4 years	(+) KOOS, IDKC, Tegner, and VAS scores were significantly improved in both the over and under 45 year-old groups. MRI determined 80% defect filling in the over 45 group and 71% defect filling in the under 45 group, and histology on 3 and 2 patients from these groups, respectively, showed good tissue repair with varying amounts of hyaline-like tissue.
	Dhollander et al., 2011 [123]	Collagen	Scaffold	PRP	2 years	(+) VAS, Tegner, Kujala patellofemoral, and KOOS scores showed improvement. MRI data showed incomplete filling in 3/5 patients, hypertrophy in 2/5 patients. Complete integration with adjacent was observed in all patients, but the surface of the repair tissue was irregular in all patients during 1 year and 2 year post-operative scans. MOCART scores remained stable during the 2 year period.
	Enea et al., 2015 [124]	Collagen	Membrane	BMAC	2 years	(+/−) Significant (*p* < 0.05) IKDC subjective score improvement, Lysholm score, VAS, and activity level pre vs. post-operative, but no change in Tegner score. MRI scans taken between 6–9mo after surgery showed reconstitution of original cartilage levels, bone marrow edema and/or subchondral; irregularities for all cases. Histologically, only 1/5 had hyaline-like matrix, and that matrix did not exhibit cell arrangements of normal articular cartilage.
	Gigante et al., 2012 [82]	Collagen	Membrane	BMAC	2 year	(+) Full weight bearing in 6 weeks, jogging at 6 months, continuously asymptomatic at 24 months. MRI scan at 12 months showed good defect filling with tissue signal signals similar to that or surrounding tissue without signs of bone marrow edema.
	Gigante et al., 2011 [125]	Collagen	Scaffold	BMAC	1 year	(+/−) All 5 patients self-reported themselves as asymptomatic. Mean histological scores of 59.8(SD 14.5). 1 had hyaline-like cartilage,3 had hyaline/fibrocartilage, and 1 had fibrocartilage formation. Columnar structures of normal articular cartilage were not observed in any case.
	Giannini et al., 2013 [126]	Collagen	Scaffold	BMAC	4 years	(+/−) AOFAS score improved significantly (*p* < 0.0005) at 24mo, but decreased significantly between 24mo and 36mo (*p* < 0.001), and 24mo to 48mo (*p* < 0.005). MRI T2 mapping analysis showed that regenerated tissue has similar values to that of hyaline cartilage −9/20 had complete defect filling and 13/20 had tissue integration at the border zone. However, a majority of patients also had damaged subchondral lamina, disrupted subchondral bone, and subchondral edema.
	Gobbi et al., 2011 [127]	Collagen	Matrix	BMAC	2 years	(+) Visual analog scale (VAS), IKDC, KOOS, Lysholm, Marx, SF-36, and Tegner scores all showed significant improvement after the final follow-up of 15 patients (*p* < 0.005). MRI T2 and histology showed hyaline like cartilage formation and complete defect filling of 12 of 15 patients with no signs of hypertrophy. Integration with adjacent cartilage was complete in 14 of those same patients.
	Krych et al., 2016 [105]	PLGA	Scaffold	PRP/BMAC	1 year	(+) No subjective clinical outcome measures were included in 11 control patients, 23 PRP treated patients, and 12 BMAC treated patients. PRP and BMAC patients both had significantly (*p* < 0.002, *p* < 0.03) better fill than the control and was more hyaline like as determined my MRI T2 mapping.
	Skowronski et al. 2013 [128]	Collagen	Membrane	BMAC	5 years	(+) An improvement was observed in 52 out of 54 patients in all scales (KOOS, Lysholm, VAS, KOOs pain) after comparison between Preoperative and 12 months post-operatively. No differences were observed between 12 months and 5 years after surgery.

(+) superior results, (−) inferior results, (=) similar results, AOFAS = American Orthopaedic Foot and Ankle Society, HA = hyaluronic acid, Hap = hydroxyapatite, IKDC = International Knee Documentation Committee, MGCSH = medical-grade calcium sulfate hemihydrate, MOCART = Magnetic Resonance Observation of Cartilage Tissue, MRI = magnetic resonance imaging, PLGA-HA = poly(glycolic acid)-hyaluronic acid, VAS = visual analog scale, KOOS = Knee injury Osteoarthritis Outcome Score, β-TCP = β-tricalcium phosphate.
